# Practices, Perceived Benefits, and Barriers Among Medical Students and Health Care Professionals Regarding the Adoption of eHealth in Clinical Settings: Cross-sectional Survey Study

**DOI:** 10.2196/34905

**Published:** 2022-09-13

**Authors:** Long Hoang Nguyen, Lien Thi Khanh Nguyen, Tham Thi Nguyen, Vu Anh Trong Dam, Thuc Minh Thi Vu, Hao Anh Si Nguyen, Giang Thu Vu, Carl A Latkin, Roger C M Ho, Cyrus S H Ho

**Affiliations:** 1 University of Medicine and Pharmacy Vietnam National University Hanoi Vietnam; 2 Institute for Preventive Medicine and Public Health Hanoi Medical University Hanoi Vietnam; 3 Institute for Global Health Innovations Duy Tan University Da Nang Vietnam; 4 Faculty of Medicine Duy Tan University Da Nang Vietnam; 5 Institute of Health Economics and Technology Hanoi Vietnam; 6 Center of Excellence in Evidence-Based Medicine Nguyen Tat Thanh University Ho Chi Minh City Vietnam; 7 Bloomberg School of Public Health Johns Hopkins University Baltimore, MD United States; 8 Department of Psychological Medicine Yong Loo Lin School of Medicine Singapore Singapore; 9 Institute for Health Innovation and Technology (iHealthtech) National University of Singapore Singapore Singapore

**Keywords:** eHealth, literacy, perception, practices, health care professional

## Abstract

**Background:**

eHealth is increasingly becoming an indispensable part of health practice and policy-making strategies. However, the use of eHealth tools in clinical practice and the perceptions of eHealth among medical students and health care professionals in Vietnam are not well understood.

**Objective:**

This study aims to investigate perceptions and practices regarding eHealth and their associated factors among medical students and health care professionals.

**Methods:**

A web-based cross-sectional study was conducted on 523 medical students and health care professionals. Information about the practices for, perceived barriers to, and benefits of eHealth application in clinical practices was collected. Multivariate Tobit and logistic regression models were used to determine factors associated with perceptions and practices.

**Results:**

In total, 61.6% (322/523) of participants used eHealth tools in clinical practices, with moderate levels of eHealth literacy. The score for the perceived benefits of eHealth tools was low. The most common barrier for eHealth utilization was human resources for IT (240/523, 45.9%), followed by security and risk control capacity (226/523, 43.2%) and no training in eHealth application (223/523, 42.6%). Age, eHealth literacy, and the use of the internet for updating medical knowledge were positively associated with using eHealth tools in clinical practices.

**Conclusions:**

eHealth tools were moderately used in clinical practices, and the benefits of eHealth were underestimated among health care professionals and medical students in Vietnam. Renovating the current medical education curriculum to integrate eHealth principles should be required to equip health care professionals and medical students with essential skills for rapid digital transformation.

## Introduction

*eHealth* refers to the use of information and communication technologies (ICTs) to improve health care, health, well-being [1]. ICTs are widely known as important tools in the health sector [2-6]. Their application is increasingly common, particularly in managing and caring for people’s health at low cost, and they have the ability to be scaled up to different settings [7-13]. eHealth tools help to ensure access to care, equity, patient-centeredness, and the quality of care [13,14]. The use of eHealth in health care not only helps health care professionals with medical examinations and treatments but also increases patients’ medication adherence and overall quality of life [15-17]. The World Health Organization reports that universal health coverage can be rapidly achieved through eHealth strategies and policies [18].

The use of eHealth tools in health care is widely available in high-income countries, such as Europe and the United States, but it is limited in resource-constrained settings. Surveys from several European countries have reported that approximately 99.7% of general practitioners use computers in clinical practice [19]. Meanwhile, in Tanzania and Ghana, only 29.4% and 60% of health care workers have ever used computers, respectively [20]. Effective eHealth adoption requires the development of an ICT system, as well as the appropriate awareness and attitudes among health care workers. To facilitate eHealth application, the increasing perceptions and practices toward eHealth among health care professionals should be given attention. Prior literature has revealed that most medical students and health care professionals have a positive outlook on eHealth [21-23]. For instance, in India, 60% of physicians have a high awareness of the benefits of adopting eHealth tools [24]. Another study in Saudi Arabia showed that about 90% of physicians agree on the benefits of eHealth [25]. In terms of medical students, prior studies have found that they have positive attitudes toward eHealth and the integration of eHealth into medical curricula [21]. A study in Austria indicated that compared to health care professionals, medical students have less belief in the usefulness of eHealth in improving patients’ knowledge but are convinced that eHealth could diminish health care costs [26]. Similarly, a study in China showed that medical students perceive more potential drawbacks with eHealth tools for telemedicine than health care professionals [27]. Several factors that determine positive perceptions and attitudes toward the use of eHealth include age, sex, living area, clinical experience, and the receipt of training for eHealth [19]. However, health care professionals have realized that there are still barriers and challenges to eHealth application in clinical clerkships, such as finance and information technology skills [28,29].

eHealth and ICT applications have been deployed in Vietnam's health sector, mainly in urban areas [30]. A national eHealth strategy was developed via a collaboration between the World Health Organization, the International Telecommunication Union, and the Ministry of Health of Vietnam. This strategy includes (1) a national eHealth vision; (2) an implementation road map for identifying key priorities in the national eHealth context; and (3) a plan for monitoring and implementing risk management, assurances with long-term investment, and support [31]. Although eHealth has been regarded as a useful tool for clinical practices, evidence about perceptions of and practices for eHealth among prospective and current medical professionals in Vietnam is scarce. A prior study in 2018 reported limited knowledge about eHealth among Vietnamese medical students, which was the result of students lacking computer skills and the intention to seek eHealth information [32]. There remains no exploration of perceptions and current practices regarding eHealth tools among health care professionals in Vietnam. This study aims to investigate perceptions and practices regarding eHealth and their associated factors among current and prospective medical professionals.

## Methods

### Study Setting and Sampling

In February 2020, we conducted a web-based cross-sectional survey among health care professionals and medical students in Vietnam. Participants were recruited if they met the following inclusion criteria: (1) living in Vietnam, (2) studying or working at hospitals or medical universities in Vietnam and having clinical experiences, (3) having either an email account or an account on social networking sites for inviting peers, and (4) providing electronic consent to participate in this study. The snowball sampling technique was used to recruit participants. Initially, a core group of health care professionals and students from three universities (Hanoi Medical University, University of Medicine and Pharmacy at Ho Chi Minh City, and Hue University of Medicine and Pharmacy) representing the three regions of Vietnam was selected for recruitment. These participants were selected due to their wide social and peer networks, which were important for the sampling technique. A web-based survey link containing a structured questionnaire was sent to the core group from the three universities via their emails. We asked participants to invite any acquaintances who met the selection criteria to participate in this web-based survey. A total of 523 health care professionals and medical students met the above criteria and were recruited in this study.

### Ethics Approval

The study protocol was approved by the Institutional Review Board of Vietnam Youth Research Institute (decision number: 177 QĐ/TWĐTN-VNCTN; date: December 28, 2018).

### Measurements

#### Overview of the Questionnaire

We designed a structured questionnaire on the SurveyMonkey platform (Momentive Inc). The contents of the questionnaire were piloted on 10 medical students and health care professionals. After revising the questionnaire based on their feedback, the final version of the questionnaire was approved and uploaded to the web-based platform. The structured questionnaire consisted of three question groups related to general socioeconomic characteristics, perceptions, and practices toward eHealth in diagnosing and treating diseases.

#### Sociodemographic Variables

The socioeconomic variables included age, living area (city or town, rural area, or mountainous area), specialty (clinical medicine or other), type of occupation (health care professionals or medical students), years of clinical experience, and city or province (Hanoi, Ho Chi Minh City, or other).

#### Internet Use Purposes and Perceived Level of eHealth Literacy

Participants were asked to report whether they used the internet to update their medical knowledge, read the news, or use social networks. Moreover, they were asked to report the frequency of using computers and smartphones for work and studies. Skills related to web-based medical document literacy were self-assessed on a 10-point scale. These included the following: identifying a medical problem, searching for medical information, evaluating the quality of a medical information source, evaluating the quality of medical information, and using medical information in clinical practice. These items had a Cronbach α value of .95, suggesting excellent internal consistency.

#### Using eHealth Tools in Clinical Practices

We asked participants to report whether they used eHealth tools in clinical practices. In this study, the use of eHealth tools was defined as the use of electronic means in consultations, examinations, diagnoses, screening, the classification of diseases, the provision of treatment regimens, and the monitoring of a patient's treatment.

#### Perceptions About Benefits of eHealth Tools

We investigated perceptions about the benefits of eHealth tools among medical professionals and medical students based on clinical practice aspects (ie, using eHealth tools to reduce medical error, to improve diagnostic quality, to improve the quality of treatment, and to provide data for clinical and public health studies), patient aspects (ie, using eHealth tools to increase patient compliance, to increase patient satisfaction, and to increase the accessibility of medical services and the benefit of eHealth for patients), and economic and organizational aspects (ie, using eHealth tools to limit unnecessary or duplicate laboratory tests or services, to increase the number of patients using daily services, to reduce costs by avoiding duplication, to increase coordination between departments in health care facilities, and to increase work productivity due to quick access to patient data).

The perception scores for the clinical practice aspects (4 items), patient aspects (3 items), and economic and organizational aspects (5 items) of the benefits of eHealth were calculated by summing the scores of all items in each domain. The scores for the three domains ranged from 0 to 4, from 0 to 3, and from 0 to 5, respectively. A higher score indicated a higher level of perceived benefits for each aspect. The Cronbach α of the scale was .87.

#### Perceptions About Barriers to Adopting eHealth Tools

We explored the perceptions of participants regarding potential barriers to applying eHealth tools in clinical practices with the following items: (1) the lack of standard procedures, (2) the lack of regulation, (3) the capacity to deploy information technology, (4) no funding, (5) security and risk control capacity, (6) not enough time, (7) difficult to use, (8) medical staff lacks information technology skills, (9) no training in eHealth application, and (10) human resources for information technology.

### Data Analysis

Both descriptive and analytical statistics were performed by using Stata 15 (StataCorp LLC). Continuous variables were presented as means and SDs, while categorical variables were presented as frequencies and percentages. We used the Wilcoxon rank-sum test for continuous variables and the chi-square test for categorical variables to compare differences between participants who were using and not using eHealth tools in clinical practices. A multivariate Tobit censored regression was performed to determine factors associated with the three domain scores and the overall score for the perceptions toward the benefits of eHealth application. Additionally, a multivariate logistic regression model was carried out to examine determinants of eHealth tool use in clinical practices. We applied a stepwise forward strategy, which involved using a log-likelihood ratio test in which the *P* value was set at .20, to select variables for the reduced models. The collinearity between variables in the model was tested by using the *collin* packages in the Stata software [33]. The number of years of clinical experience was found to have collinearity with age; thus, we excluded the years of clinical experience variable. Afterward, the variance inflation factor of predictor variables was less than 10, and the average of the variance inflation factor was 3.6, suggesting that there was no collinearity. The statistical significance was set at an α level of .05.

## Results

Table 1 shows that a total of 91.2% (476/522) of the recruited participants were medical students. Over 70% (367/523, 70.2%) of participants were female, 90.1% (471/523) of participants lived in urban areas, and more than half of the participants (268/523, 51.2%) were specializing in clinical medicine. The mean age was 21.7 (SD 4.5) years, and the mean number of years of clinical experience was 3.7 (SD 4.5). Further, 61.6% (322/523) of participants reported that they have used eHealth tools for clinical practice. There was a significant difference in the usage of eHealth tools by sex (*P*=.02), specialty (*P*<.001), the number of years of clinical experience (*P*<.001), and the type of occupation (*P*<.001). Table 1 also shows that 40.2% (210/523) of the sample used the internet for updating their medical knowledge. The proportion of participants who were using computers and smartphones for work and studies regularly was 78.8% (406/515). Participants showed a moderate level of skills for searching, evaluating, and using medical documents on the internet, and participants who had ever used eHealth tools in clinical practices gave significantly higher scores than those given by participants who were not using eHealth tools.

**Table 1:** Characteristics of the respondents.

Characteristics		Using eHealth tools for clinical practice				*P* value	
		No^a^	Yes^a^	Total^b^			
Participants, n (%)		201 (38.4)	322 (61.6)	523 (100)	N/A^c^		
**Sex, n (%)**							.02
	Male	48 (30.8)	108 (69.2)	156 (29.8)			
	Female	153 (41.7)	214 (58.3)	367 (70.2)			
**Specialty, n (%)**							<.001
	General practitioner	83 (31)	185 (69)	268 (51.2)			
	Other	118 (46.3)	137 (53.7)	255 (48.8)			
**Type of occupation, n (%)**							<.001
	Health care professionals	5 (10.9)	41 (89.1)	46 (8.8)			
	Medical students	196 (41.2)	280 (58.8)	476 (91.2)			
**Living area, n (%)**							.77
	City	182 (38.6)	289 (61.4)	471 (90.1)			
	Town, rural area, or mountainous area	19 (36.5)	33 (63.5)	52 (9.9)			
**Region, n (%)**							.39
	Northern region	53 (41.4)	75 (58.6)	128 (25.3)			
	Southern region	120 (39.6)	183 (60.4)	303 (59.9)			
	Central region	24 (32)	51 (68)	75 (14.8)			
**Purpose of using the internet, n (%)**							
	To update medical knowledge	53 (25.2)	157 (74.8)	210 (40.2)	<.001		
	To read the news	106 (36.3)	186 (63.7)	292 (55.8)	.26		
	To use social networks	174 (39.6)	265 (60.4)	439 (83.9)	.20		
**Frequency of using computers or smartphones for work or studies, n (%)**							.055
	Yes, regularly	149 (36.7)	257 (63.3)	406 (78.8)			
	Yes, sometimes	51 (46.8)	58 (53.2)	109 (21.2)			
Age (years), mean (SD)		20.7 (2.8)	22.3 (5.2)	21.7 (4.5)	<.001		
Years of clinical experience, mean (SD)		2.7 (2.8)	4.3 (5.2)	3.7 (4.5)	<.001		
**Perceived levels of eHealth literacy (score; range 0-10), mean (SD)**							
	Using eHealth tools to identify a problem	6.0 (2.2)	6.8 (1.8)	6.5 (2.0)	<.001		
	Using eHealth tools to search for medical information	6.2 (2.0)	6.7 (1.9)	6.5 (2.0)	.003		
	Using eHealth tools to evaluate the quality of a medical information source	5.6 (2.2)	6.4 (2.0)	6.1 (2.1)	<.001		
	Using eHealth tools to evaluate the quality of medical information	5.7 (2.2)	6.4 (1.9)	6.1 (2.0)	<.001		
	Using eHealth tools to use medical information in clinical practice	5.5 (2.3)	6.4 (2.0)	6.0 (2.2)	<.001		

^a^Percentages in this column were calculating by using the *Total* column value as the denominator.

^b^The totals do not add up to 523 throughout this column due to missing or multiple responses.

^c^N/A: not applicable.

Table 2 shows that the benefits of eHealth tools were perceived by both groups equally (ie, all *P* values are >.05). With regard to organizational and economical aspects, increased work productivity due to quick access to patient data was the most common perceived benefit (314/523, 60%), followed by increased coordination between departments in health facilities (301/523, 57.6%). In terms of clinical practice aspects, the proportion of participants who perceived the benefit that eHealth tools provide data for clinical and public health studies was the highest (33/523, 63.1%), followed by the proportion who perceived that eHealth tools improve diagnostic quality (283/523, 54.1%). With regard to patient aspects, the most common benefit was increasing the accessibility of medical services for patients (267/523, 51.1%). However, overall, participants believed that using eHealth tools was not quite beneficial in clinical settings (score out of 12: mean 5.4, SD 3.6).

Table 3 shows potential barriers for eHealth application. The most common barrier was human resources for IT (240/523, 45.9%), followed by security and risk control capacity (226/523, 43.2%) and no training in eHealth application (223/523, 42.6%). There were no differences in the perceived barriers between participants who were using and not using eHealth tools in clinical practices (ie, all *P* values are >.05; Table 3).

Table 4 presents the factors associated with the three perception domain scores and the use of eHealth tools in clinical practice. There was a positive correlation between age and the use of eHealth tools for clinical practice (odds ratio 1.09, 95% CI 1.02-1.18). The use of the internet to update medical knowledge and higher scores for identifying a problem in web-based documents were associated with a higher likelihood of using eHealth tools for clinical practice.

Female participants had significantly lower scores for the perceptions regarding the patient-related aspects of eHealth compared to those of male participants (coefficient=−0.45, 95% CI −0.88 to −0.02; *P*=.04). Medical students had lower scores compared to those of health care professionals for the perceptions regarding the clinical aspects (coefficient=−0.94, 95% CI −1.78 to −0.10; *P*=.004) and organization and economic aspects (coefficient=−1.40, 95% CI −2.19 to −0.61; *P*=.001) of eHealth usage. Using the internet to update medical knowledge, read the news, and use social networks was associated with higher perceptions regarding clinical practice aspects, and using the internet to read the news was also positively related to higher perceptions about the organization and economic aspects of eHealth usage (coefficient=0.65, 95% CI 0.21-1.09; *P*=.004). Perceptions about the patient aspects of eHealth use positively correlated with the perceived levels for the evaluation of an information source (coefficient=0.40, 95% CI 0.16-0.65; *P*=.001) but negatively correlated with the perceived levels for the evaluation of information (coefficient=−0.35; 95% CI −0.60 to −0.10; *P*=.006). Perceptions about the organization and economic aspects of eHealth positively correlated with the perceived levels for identifying a problem (coefficient=0.14, 95% CI 0.03-0.25; *P*=.02).

**Table 2:** Perceptions on the use of eHealth.

Perceptions about benefits of eHealth tools			Using eHealth tools for clinical practice							*P* value
			No (n=201)		Yes (n=322)		Total (N=523)			
**Organizational and economical aspects, n (%)**										
	Using eHealth tools to increase work productivity due to quick access to patient data	120 (59.7)		194 (60.2)		314 (60)		.90		
	Using eHealth tools to increase coordination between departments in health facilities	114 (56.7)		187 (58.1)		301 (57.6)		.76		
	Using eHealth tools to reduce costs by avoiding duplication	69 (34.3)		129 (40.1)		198 (37.9)		.19		
	Using eHealth tools to increase the number of patients using daily services	58 (28.9)		100 (31.1)		158 (30.2)		.59		
	Using eHealth tools to limit unnecessary or duplicate laboratory tests or services	63 (31.3)		127 (39.4)		190 (36.3)		.06		
**Clinical practice aspects, n (%)**										
	Using eHealth tools to provide data for clinical and public health studies	122 (60.7)		208 (64.6)		330 (63.1)		.37		
	Using eHealth tools to improve the quality of treatment	89 (44.3)		140 (43.5)		229 (43.8)		.86		
	Using eHealth tools to improve diagnostic quality	107 (53.2)		176 (54.7)		283 (54.1)		.75		
	Using eHealth tools to reduce medical error	93 (46.3)		162 (50.3)		255 (48.8)		.37		
**Patient aspects, n (%)**										
	Using eHealth tools to increase patients’ access to medical services	103 (51.2)		164 (50.9)		267 (51.1)		.95		
	Using eHealth tools to increase patient satisfaction	67 (33.3)		111 (34.5)		178 (34)		.79		
	Using eHealth tools to increase patient compliance	37 (18.4)		72 (22.4)		109 (20.8)		.28		
Organizational and economical aspects score (range 0-5), mean (SD)			2.1 (1.6)		2.3 (1.7)		2.2 (1.7)		.28	
Clinical aspects score (range 0-4), mean (SD)			2.0 (1.5)		2.1 (1.5)		2.1 (1.5)		.52	
Patient aspects score (range 0-3), mean (SD)			1.0 (1.0)		1.1 (1.1)		1.1 (1.1)		.76	
Total score (range 0-12), mean (SD)			5.2 (3.6)		5.5 (3.5)		5.4 (3.6)		.28	

**Table 3:** Barriers for eHealth application.

Barriers		Using eHealth tools for clinical practice, n (%)				*P* value
		No (n=201)	Yes (n=322)	Total (N=523)		
**Organizational and economical barriers**						
	Lack of standard procedure	51 (25.4)	103 (32)	154 (29.4)	.11	
	Lack of regulation	66 (32.8)	106 (32.9)	172 (32.9)	.98	
	The capacity to deploy IT	72 (35.8)	128 (39.8)	200 (38.2)	.37	
	No funding	76 (37.8)	141 (43.8)	217 (41.5)	.18	
	Security and risk control capacity	96 (47.8)	130 (40.4)	226 (43.2)	.10	
**Clinical and technical barriers**						
	Not enough time	25 (12.4)	36 (11.2)	61 (11.7)	.66	
	Difficult to use	33 (16.4)	54 (16.8)	87 (16.6)	.92	
	Medical staff lacks IT skills	83 (41.3)	124 (38.5)	207 (39.6)	.53	
	No training in eHealth application	89 (44.3)	134 (41.6)	223 (42.6)	.55	
	Human resources for IT	98 (48.8)	142 (44.1)	240 (45.9)	.30	

**Table 4:** Factors associated with practice and positive perceptions.

Variables			Using eHealth tools for clinical practice, OR^a^ (95% CI)		Perceptions about the use of eHealth, coefficient (95% CI)				
					Clinical aspects		Patient-related aspects		Organization and economic aspects
Age (per year)			1.09^b^ (1.02 to 1.18)		N/A^c^		N/A		N/A
**Sex**									
	Male	N/A		N/A		Reference		N/A	
	Female	N/A		N/A		−0.45^b^ (−0.88 to −0.02)		N/A	
**Specialty**									
	Clinical medicine	Reference		N/A		N/A		N/A	
	Other	0.64^d^ (0.43 to 0.97)		N/A		N/A		N/A	
**Type of occupation**									
	Health care professionals	N/A		Reference		N/A		Reference	
	Medical students	N/A		−0.94^b^ (**−**1.78 to **−**0.10)		N/A		−1.40^d^ (**−**2.19 to **−**0.61)	
**Purpose of using the internet**									
	Update medical knowledge (yes vs no)	2.24^d^ (1.45 to 3.46)		0.69^d^ (0.20 to 1.19)		N/A		N/A	
	Read the news (yes vs no)	N/A		0.64^d^ (0.17 to 1.12)		0.34^e^ (**−**0.06 to 0.74)		0.65^d^ (0.21 to 1.09)	
	Social networks (yes vs no)	0.68 (0.39 to 1.19)		1.00^d^ (0.40 to1.60)		N/A		0.49^e^ (**−**0.09 to 1.07)	
**Perceived levels of eHealth literacy**									
	Using eHealth tools to identify a medical problem (per point)	1.20^d^ (1.08 to 1.33)		N/A		N/A		0.14^b^ (0.03 to 0.25)	
	Using eHealth tools to evaluate the quality of a medical information source (per point)	N/A		N/A		0.40^d^ (0.16 to 0.65)		N/A	
	Using eHealth tools to evaluate the quality of medical information (per point)	N/A		N/A		−0.35^d^ (−0.60 to −0.10)		N/A	

^a^OR: odds ratio.

^b^Significant at the *P*<.05 level.

^c^N/A: not applicable.

^d^Significant at the *P*<.01 level.

^e^Significant at the *P*<.10 level.

## Discussion

### Principal Findings

Health technology and eHealth have been becoming indispensable components in hospital operation and patient care. This study contributed to the current literature to facilitate the use of eHealth principles in Vietnamese clinical settings. Our findings indicated that eHealth tools were widely used among the health care professionals, but only more than half of the medical students (280/476, 58.8%) frequently used these tools in their clinical practices. Perceived benefits and barriers in using eHealth were also explored, and the results of the multivariate analysis indicated further implications for facilitating the use of eHealth in clinical practices.

Promoting the development of the eHealth system in Vietnam plays an important role in improving the quality of patient care and hospital efficiency [34]. Current eHealth systems are being applied in Vietnam, such as telemedicine systems that help support patients remotely during the COVID-19 pandemic; artificial intelligence systems that help diagnose cancer and lung diseases; and other eHealth systems, including an eHealth book system that helps manage disease status [35-37]. Especially in the context of emergency events and disasters, such as the COVID-19 pandemic, the use of eHealth tools has become even more urgent [38]. This requires current physicians and medical students to be fully equipped with sufficient eHealth literacy, which is needed to adapt to the increasing demands of these systems.

The use of eHealth tools in clinical settings was commonly observed among health care professionals but was still limited among medical students. However, our proportion of participants who were using eHealth tools was higher than that of a 2017 study in China, which revealed that only 51.1% of health care professionals and 41.6% of medical students had heard of telehealth [27]. Prior research in Tanzania and Ghana found that only 29.4% and 60% of health care workers have ever used computers, respectively [20]. Another study in the United States showed that merely 17.4% of medical students had experience with telemedicine [39]. Our results were understandable since, in recent years, the advancement and popularity of the internet and electronic devices (eg, laptops, smartphones, or tablets) has increasingly allowed health care professionals and medical students to conveniently access a variety of eHealth tools that are available on the internet. Moreover, the national strategy on eHealth has promoted the use of eHealth tools in clinical settings, which provides opportunities for these groups to approach and use such tools. Nonetheless, compared to findings from European countries, where 99.7% of practitioners use computers in clinical practice [19], the proportion of participants who were using eHealth tools in our study was considerably lower, suggesting that there is a huge gap that needs to be filled for the success of digital transformation in health care.

The results of this study show that the participants' levels of eHealth literacy were moderate. Given the nature of eHealth tools and systems to be innovative and to change continuously, these results indicate a potential barrier to the use and adaptation of eHealth tools [40]. Indeed, nearly half of the participants found a lack of ICT skills (207/523, 39.6%) and a lack of training (223/523, 42.6%) to be considerable challenges to using eHealth tools in clinical practices. This phenomenon could be explained by the fact that eHealth capabilities have not been systematically integrated into the current undergraduate and graduate medical curricula in Vietnam and only appear in several continuing medical education training programs. This gap can become serious if the curricula are not reformed, due to the rapid development of medical technology. Providing the most foundational eHealth skills to medical students and medical practitioners will help them adapt to the digital transformation and proficiently use eHealth tools to serve their practices [34,40].

The findings of this study also show that a great barrier to the application of eHealth in Vietnam was that physicians and medical students did not recognize the roles and benefits of eHealth tools in clinical practices. Specifically, the scores for the perceived benefits of eHealth tools were below moderate, suggesting that the benefits of eHealth for participants were not quite clear. This issue might be justified by the fact that although a national eHealth strategy had been proposed and implemented, eHealth systems in hospitals at the time of this study were still in their beginning stages, despite the major presence of the eHealth management system. Only a few central hospitals and private hospitals adopt advanced eHealth systems, such as artificial intelligence systems. Therefore, it is understandable that the physicians and medical students, particularly the latter, did not have much exposure to eHealth tools and were not fully aware of the role of eHealth. However, the COVID-19 pandemic has accelerated the digital transformation process in all levels of the Vietnamese health care system [38]. Further, all hospitals benefited from this innovation. These benefits included the implementation of telemedicine and remote disease management and diagnosis, which fostered cooperation and technology transfer between central hospitals and primary health care facilities [37,41]. Therefore, it is expected that the perceptions of health care professionals will change and that they will quickly prepare for the process of adapting to future eHealth technologies.

In our study, medical students had lower scores for the perceptions about the organization and economic aspects and clinical aspects of the usefulness of eHealth. This study was different from a study in Austria, wherein the authors found that medical students were more optimistic about the use of eHealth to reduce health care costs but more pessimistic about the use of eHealth to improve patients’ knowledge when compared to health care professionals [26]. Another study in China reported that medical students have more concerns about telehealth than health care professionals, which might be due to their low awareness and utilization of telehealth [27]. A study in the United States found that increasing exposure to telemedicine could raise the awareness and attitudes of medical students regarding telemedicine [39]. Given their bridging role between health care professionals and patients, medical students are suggested to have more positive views of eHealth application than those of other groups. Moreover, medical schools and hospitals should offer more opportunities to medical students that expose them to eHealth tools in clinical settings. This might improve their opinions about eHealth and provide them with the capacity to perform clinical practices in the future.

This study demonstrates the important role of systematically building and integrating eHealth capacities into current medical training curricula. This would be useful for physicians and medical students who can adapt to the great digital transformation of health care in Vietnam. These individuals may have good ICT skills, but they may also have limitations in evaluating the medical information they find and using medical information in clinical practice. Further studies on the development of practical training frameworks for eHealth techniques that narrow the gaps between academia and reality should also be considered and implemented.

This study has several limitations that need to be considered when interpreting the results. First, our cross-sectional survey was based on self-reported information, which might result in recall bias. Second, this study had the limitations of a cross-sectional design, which did not allow us to draw causal relationships between eHealth practices, perceived benefits of eHealth, and associated factors. Third, the snowball sampling method limited the generalizability of the study results to health care professionals and medical students in Vietnam. Fourth, the sample of health care professionals was small. To develop a full picture of the perceptions and practices regarding the application of eHealth in diagnosis and treatment among health care workers in Vietnam, additional studies should be conducted with larger sample sizes. Moreover, qualitative research should be performed to more comprehensively understand the perceptions of these populations regarding the use of eHealth tools.

### Conclusion

This paper informs that in Vietnam, eHealth tools are moderately used in clinical practices, and the benefits of eHealth are underestimated among health care professionals and medical students. Renovating the current medical education curriculum to integrate eHealth principles should be required to equip health care professionals and medical students with essential skills for rapid digital transformation.

## Abbreviations

ICT: information and communication technology
